# Lymphatic and Glymphatic Alterations in Auditory Disorders: A Rapid Review-Informed Systematic Review and Meta-Analysis

**DOI:** 10.3390/medicina62050878

**Published:** 2026-05-03

**Authors:** Andrea Frosolini, Paolo Gennaro

**Affiliations:** Maxillofacial Surgery Unit, Department of Medical Biotechnology, S. Maria alle Scotte University Hospital of Siena, 53100 Siena, Italy; gennaro2@unisi.it

**Keywords:** glymphatic system, inner ear, lymphatic vessels, DTI-ALPS, magnetic resonance imaging, age-related hearing loss, tinnitus, cognitive decline

## Abstract

*Background and Objectives*: The inner ear has traditionally been regarded as an immunoprivileged and anatomically isolated organ. However, growing interest in neuro-lymphatic interactions has raised the hypothesis that glymphatic and lymphatic mechanisms may contribute to auditory pathology and its association with cognitive dysfunction. This systematic review aimed to synthesize current human evidence regarding anatomical, imaging, and clinical correlates of glymphatic mechanisms in the inner ear and audiological pathologies, and to quantitatively evaluate currently available biomarkers. *Materials and Methods:* A structured search of PubMed, Scopus, and Cochrane databases was performed from inception through March 2026. Eligible studies included human investigations reporting anatomical, histopathological, or MRI-based glymphatic assessments related to inner ear disorders. Risk of bias was assessed using the Newcastle–Ottawa Scale and Joanna Briggs Institute tools. Meta-analysis was conducted for diffusion tensor image analysis along the perivascular space (DTI-ALPS) indices comparing auditory disorders with healthy controls. *Results*: Six studies met inclusion criteria (five cross-sectional imaging studies and one surgical histopathological case series). Histopathology demonstrated lymphatic capillaries in advanced Ménière disease. MRI studies consistently reported reduced ALPS indices and/or increased choroid plexus volume and enlarged perivascular spaces in tinnitus, congenital sensorineural hearing loss, and age-related hearing loss. Meta-analysis of five studies showed a significant reduction of ALPS index in auditory disorders compared with controls (SMD = −0.73, 95% CI −0.90 to −0.55; *p* < 0.001), with no heterogeneity. Glymphatic markers were frequently associated with audiological data, cognitive performance and inflammatory biomarkers. *Conclusions*: Human evidence supports the presence of altered central glymphatic function across diverse auditory phenotypes. Although predominantly based on indirect MRI proxies and cross-sectional data, the meta-analytic findings strengthen the biological plausibility of an auditory–glymphatic interaction. Prospective longitudinal studies are warranted to clarify causality and therapeutic implications.

## 1. Introduction

For decades, the inner ear has been considered a relatively immunoprivileged organ, protected by the blood–labyrinth barrier. However, since the 1970s, the paradigm of strict immunoprivilege has progressively been challenged across multiple organs and systems. Early experimental work provided the first indication that the inner ear and even the brain might not be anatomically and functionally isolated from systemic immune and lymphatic circulation [1]. In a seminal study, Yimtae et al. (2001) demonstrated a direct connection between the inner ear and the cervical lymphatic system in guinea pigs [2]. Afterwards, a major conceptual advance in understanding fluid circulation in the central nervous system came with the description of the glymphatic system by Iliff et al. (2012): A paravascular pathway that facilitates cerebrospinal fluid influx, interstitial solute clearance and interaction with astroglial water channels [3].

Technological advancements—particularly in magnetic resonance imaging (MRI)—have significantly expanded the ability to investigate fluid dynamics in vivo. High-resolution and delayed contrast-enhanced MRI techniques have enabled visualization of endolymphatic and perilymphatic compartments and have provided indirect markers of glymphatic activity in humans. Naganawa et al. (2024) summarized current MRI-based investigations of the human glymphatic system, highlighting the translational potential of imaging biomarkers in disorders characterized by altered fluid homeostasis [4]. These developments are especially relevant for inner ear research, where non-invasive assessment of labyrinthine fluid exchange was historically limited.

Recent pathological findings have further reinforced the relevance of lymphatic and glymphatic mechanisms. In patients with Ménière disease, Zhang et al. (2024) reported the presence of lymphatic vessels in the inner ear, suggesting a possible role of lymphatic dysfunction in endolymphatic hydrops [5]. Parallel research has explored the interaction between hearing ability, inflammation, and glymphatic efficiency [6]. Beyond diagnostic implications, emerging surgical and translational research has begun exploring whether modulation of lymphatic pathways may influence neurodegenerative processes. Xie et al. (2025) preliminarily demonstrated the potential role of lymphovenous bypass procedures in treating Alzheimer’s disease symptoms [7].

Despite growing interest, the role of the inner ear within lymphatic and glymphatic pathways remains incompletely defined across experimental, imaging, and clinical studies, with absence of structured literature synthesis. Given the translational implications for Ménière disease, sensorineural hearing loss, chronic tinnitus, and potentially cognitive decline, a focused evaluation of the available human evidence is warranted. Therefore, the aim of this systematic review is to synthesize current evidence regarding the existence, imaging characterization, and pathological relevance of glymphatic and lymphatic mechanisms in the human inner ear, identify knowledge gaps, and outline future directions for translational and clinical research.

## 2. Materials and Methods

This study was conducted as a rapid systematic review, designed to provide a structured and timely synthesis of the available human evidence regarding glymphatic and lymphatic mechanisms in the inner ear. The methodology followed core principles of systematic review (structured multi-database searching, predefined eligibility criteria, independent title/abstract screening, full-text assessment, standardized data extraction, and formal risk-of-bias assessment) adopting methodological streamlining consistent with rapid review approaches [8]. The review was conducted in accordance with PRISMA 2020 guidelines, and the completed checklist is provided in the Supplementary Materials. A formal protocol was not prepared and the work was not prospectively registered in a public database due to the expedited methodological framework adopted for rapid evidence synthesis [8] We acknowledge that the lack of prospective registration may reduce transparency and reproducibility and may increase the risk of selective reporting at the review level. Nonetheless, this expedited approach was adopted since the available literature—while limited and heterogeneous—is rapidly emergent, and the authors aimed to provide a timely structured synthesis of the evidence preserving the main methodology of systematic review [8]. The primary research question was: What evidence supports the presence of lymphatic or glymphatic mechanisms in the human inner ear and auditory pathway?

### 2.1. Inclusion and Exclusion Criteria

Studies were eligible if they: (i) Included human subjects (in vivo or ex vivo human tissue); (ii) Investigated glymphatic pathways and/or lymphatic vessels related to the inner ear physiology/pathology; (iii) Reported anatomical, radiological, histopathological, or clinical findings; (iv) Were original research articles; (v) Were published in peer-reviewed journals. Exclusion criteria were the following: (i) Purely animal studies (unless directly translational with human validation); (ii) Reviews and editorials; (iii) Studies addressing central glymphatic function without specific reference to the inner ear and/or auditory pathway; (iv) Non-English publications where full text was unavailable.

### 2.2. Search Strategy, Study Selection, and Data Extraction

A structured search was performed in PubMed, Scopus, and Cochrane databases. The search covered studies published from database inception through March 2026. The PubMed search strategy was based on the following combination: (“glymphatic system” OR “lymphatic vessels” OR “Lymphatic”) AND (inner ear OR hearing loss OR tinnitus). Equivalent adaptations were used for Scopus and Cochrane. Reference lists of included articles were manually screened to identify additional relevant studies. Two reviewers independently screened titles and abstracts for eligibility. Full-text assessment was then performed for potentially relevant articles. Disagreements were resolved by consensus discussion. Data were extracted using a predefined standardized form.

### 2.3. Outcome Definition, Risk of Bias Assessment, and Data Synthesis

As primary outcome we set evidence supporting the presence or functional relevance of glymphatic or lymphatic pathways in the human inner ear. As secondary outcomes we considered: (i) Imaging biomarkers suggestive of glymphatic activity; (ii) Pathological confirmation of lymphatic vessels; (iii) Associations with specific diseases (e.g., Ménière disease, sudden sensorineural hearing loss, tinnitus, cognitive impairment); (iv) Translational or surgical implications. Methodological quality was assessed using appropriate tools such as the Newcastle–Ottawa Scale (NOS) for observational studies and the Joanna Briggs Institute (JBI) checklist for case series. Item-level assessments were first performed for each included study. Overall risk of bias was then judged descriptively by considering both the number and the relevance of unmet criteria; complete item-level assessments are provided in the Supplementary Materials.

A meta-analysis was undertaken when datasets were comparable; otherwise, results were synthesized narratively. Given the anticipated clinical heterogeneity of auditory phenotypes, the meta-analysis was intended to evaluate the direction and consistency of alterations across eligible human studies rather than to infer a disease-specific common mechanism. Meta-analysis was conducted in MetaAnalysisOnline.com [9] using an inverse-variance random-effects model (DerSimonian–Laird τ^2^ estimator), with effect sizes expressed as standardized mean differences (SMD) and 95% confidence intervals (CIs). Small-study effects/publication bias were assessed using funnel plot inspection, Egger’s regression test, Begg’s rank correlation test, and trim-and-fill. The certainty of evidence for the quantitative outcome was assessed using the Grading of Recommendations Assessment, Development and Evaluation (GRADE) approach [10].

## 3. Results

The systematic search yielded a total of 728 records, including 212 from PubMed, 490 from Scopus, and 26 from Cochrane. After removal of duplicate records, 522 unique studies remained. Following title and abstract screening, 21 articles were retrieved for full-text assessment. Of these, 15 studies were excluded for the following reasons: (i) Review articles without primary human data [4,11,12,13]; (ii) Animal-only experimental studies [2,14,15,16]; (iii) Immune studies without direct lymphatic/glymphatic structural evidence [17,18,19]; (iv) Clinical or imaging studies without direct lymphatic/glymphatic assessment [20]; (v) Imaging studies not meeting final eligibility criteria [21,22,23].

**Figure 1:**
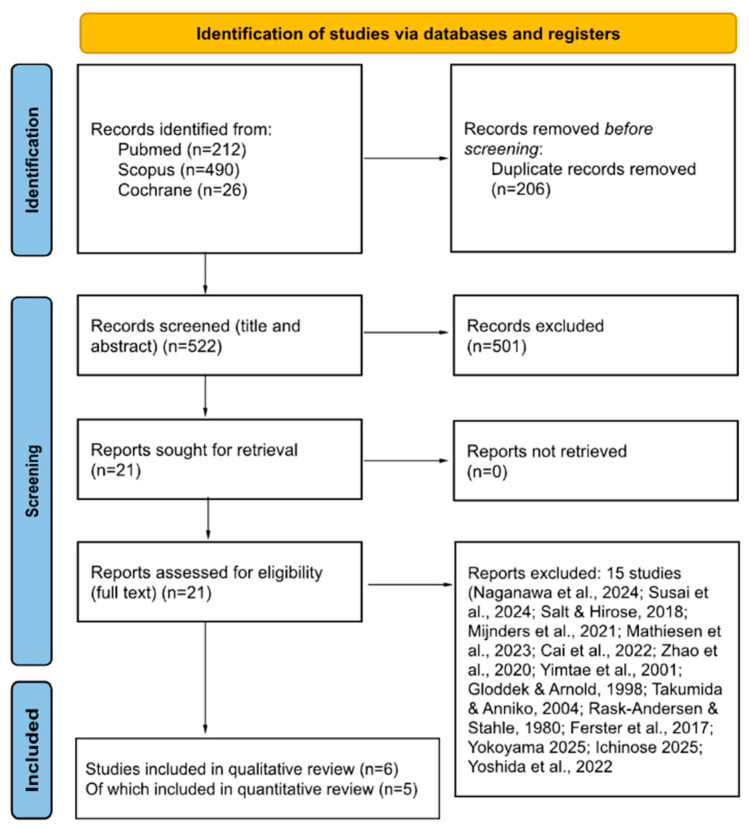
PRISMA flow diagram from database search to manuscript inclusion [2,4,11,12,13,14,15,16,17,18,19,20,21,22,23].

After full-text assessment, six studies met all predefined inclusion criteria and were included in the qualitative synthesis [5,6,24,25,26,27]. A PRISMA flow diagram summarizing the selection process is presented in Figure 1.

### 3.1. Study Characteristics and Risk of Bias

The included studies demonstrated low to moderate risk of bias, encompassing surgical histopathology case series and cross-sectional observational designs. The main characteristics and risk of bias assessment of the included studies are summarized in Table 1.

### 3.2. Evidence Synthesis According to Population, Intervention, Comparison and Outcome

The six included studies investigated distinct but partially overlapping clinical populations, specifically pediatric sensorineural hearing loss (SNHL) [27], Ménière disease (MD) [5], age-related hearing loss (ARHL) [6,26], and chronic tinnitus [24,25]. Each cohort was recruited using structured audiological assessment and predefined inclusion and exclusion criteria. Methodologically, five of the six studies relied primarily on MRI approaches, employing indirect glymphatic biomarkers such as choroid plexus volume (CPV) [6,25], enlarged perivascular spaces (EPVS) [6,25], and diffusion tensor image analysis along the perivascular space (DTI-ALPS) [24,25,26,27]. In contrast, only one study provided direct histopathological evidence, identifying lymphatic capillaries in surgical round window specimens from patients with advanced MD [5]. Across imaging-based investigations, findings were directionally consistent toward altered central glymphatic indices. Untreated pediatric SNHL, tinnitus, and ARHL cohorts demonstrated significantly reduced ALPS index values compared with controls [24,25,26,27], while multimodal studies additionally reported increased CPV and EPVS [6,25]. Cognitive assessment was performed in four imaging studies [6,24,25,26] and frequently correlated with glymphatic biomarkers and audiological measures. In chronic tinnitus, reduced ALPS index correlated with poorer executive performance (Trail Making Test-B; r = −0.309, *p* = 0.039) and verbal learning performance (r = −0.413, *p* = 0.005) [24]. In tinnitus with sleep disturbance, ALPS reduction correlated with higher Pittsburgh Sleep Quality Index (r = −0.428, *p* = 0.001) and Tinnitus Handicap Questionnaire scores (r = −0.378, *p* = 0.005) [25]. In ARHL, ALPS index was positively associated with Montreal Cognitive Assessment (MoCA; ρ = 0.426, *p* = 0.026) [26]. Furthermore, higher CPV and EPVS and lower ALPS values were significantly associated with elevated inflammatory cytokines (TNF-α, IL-1β, IL-6) [6]. Ye et al. confirmed that glymphatic indices mediated associations between hearing loss severity and cognition [6]: mean pure tone average (PTA) correlated positively with CPV (r = 0.19, q = 0.014) and EPVS (r = 0.23, q = 0.002), and negatively with ALPS (r = −0.33, q < 0.0001). ALPS was positively associated with MoCA (r = 0.17, q = 0.034), whereas CPV was negatively associated with Digit Symbol Substitution Test (DSST) scores (r = −0.19, q = 0.015). Canonical correlation analysis demonstrated significant multivariate associations between glymphatic markers and cognition (Rc = 0.375, *p* = 0.002), and between glymphatic markers and inflammatory factors (Rc = 0.505, *p* < 0.0001). Mediation analyses showed that ALPS significantly mediated the PTA–MoCA relationship (indirect effect = −0.0053, 95% CI −0.0115 to −0.0001), and CPV mediated the PTA–DSST relationship (indirect effect = −0.0048, 95% CI −0.0094 to −0.0013) [6]. In pediatric congenital SNHL, ALPS index showed a significant negative correlation with age (ρ = −0.544, *p* = 0.005) [27], suggesting early vulnerability of glymphatic activity in developmental auditory deprivation. The PICO-based synthesis of these studies is summarized in Table 2.

### 3.3. Meta-Analysis of MRI Values

**Figure 2:**
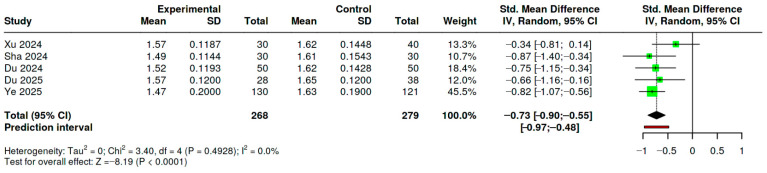
Forest Plot of Standardized Mean Differences in ALPS Index Between Auditory Disorders and Healthy Controls [6,24,25,26,27].

**Figure 3:**
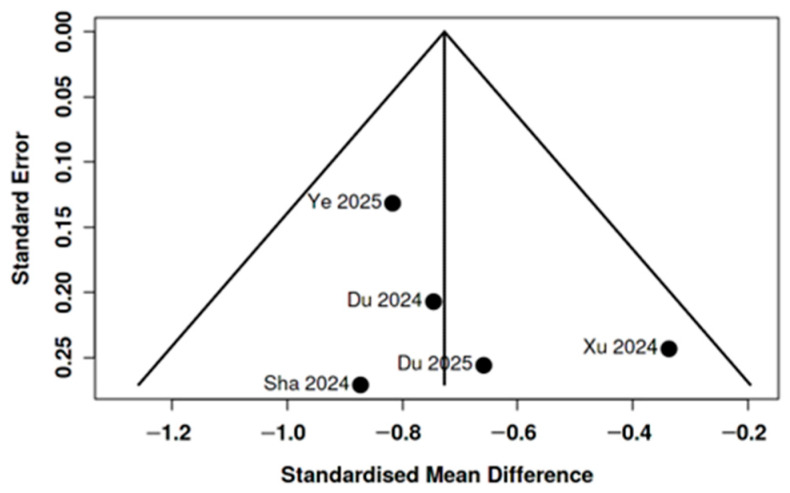
Funnel plot of the five studies included in the meta-analysis of DTI-ALPS index differences between auditory disorder groups and healthy controls: visual inspection did not suggest marked asymmetry [6,24,25,26,27].

Five cross-sectional studies reporting DTI-ALPS index values in patients with auditory disorders and healthy controls were included in a random-effects meta-analysis [6,24,25,26,27]. The pooled effect demonstrated a significant reduction in ALPS index among patients compared with controls (SMD = −0.73, 95% CI −0.90 to −0.55; z = −8.19, *p* < 0.001). Individual study estimates ranged from −0.87 to −0.34 and consistently favored lower ALPS values in affected populations (see Figure 2). No significant heterogeneity was detected (Q(4) = 3.40, *p* = 0.49; τ^2^ = 0.00; I^2^ = 0%), indicating highly consistent effect sizes across studies. Funnel plot inspection did not suggest small-study effects (see Figure 3), and Egger’s regression test was not significant (intercept 1.36, 95% CI −1.52 to 4.24; *p* = 0.423). Additional analyses did not suggest relevant publication bias or small-study effects: Begg and Mazumdar’s rank correlation test was non-significant (τ = 0.200, *p* = 0.817); Trim-and-fill analysis did not identify potentially missing studies. Overall, the quantitative synthesis indicates a moderate and directionally consistent reduction in DTI-ALPS index across heterogeneous auditory phenotypes, since the pooled populations differ substantially in age and clinical presentation.

Random-effects meta-analysis using an inverse-variance model (DerSimonian–Laird estimator) of five cross-sectional studies comparing DTI-ALPS index values between patients with auditory disorders and healthy controls. Squares represent individual study effect sizes (standardized mean differences, SMD) with 95% confidence intervals; the diamond indicates the pooled estimate (SMD = −0.73, 95% CI −0.90 to −0.55). Negative values indicate lower ALPS index in patient groups.

Sensitivity analyses confirmed the robustness of the pooled effect. Exclusion of one study [6] (k = 4) yielded a comparable standardized mean difference (SMD = −0.65, 95% CI −0.89 to −0.42; I^2^ = 0%); further restriction [25] to three studies resulted in a pooled SMD of −0.61 (95% CI −0.91 to −0.30), with low heterogeneity (I^2^ = 11.7%). A sensitivity analysis excluding the pediatric cohort [27] yielded a comparable pooled effect (k = 4; SMD = −0.70, 95% CI −0.90 to −0.51; I^2^ = 5.5%). Using GRADE, the certainty of evidence for the pooled DTI-ALPS reduction in auditory disorders was rated low, as resembled in Table 3.

## 4. Discussion

Although theoretical interest in the neuro-auditory–glymphatic interface has expanded substantially in recent years, only six studies met our inclusion criteria, underscoring the early stage of clinical investigation in this field. The available evidence encompasses two complementary domains: structural histopathological data from inner ear biopsies [5] and neuroimaging studies reporting markers of central glymphatic dysfunction [6,24,25,26,27]. The predominance of MRI-based methodologies—particularly DTI-ALPS indices and volumetric markers such as EPVS and CPV—reflects a technological shift toward non-invasive assessment of brain fluid dynamics. However, this trend also highlights a fundamental limitation: current evidence relies largely on indirect imaging biomarkers rather than direct visualization of lymphatic pathways.

The interpretation of DTI-ALPS findings is also limited by methodological variability. Manual ROI placement may introduce subjectivity and observer-dependent variation, but reproducibility studies indicate that acquisition factors such as imaging plane, head position, number of gradient axes, and echo time may exert an even greater influence on ALPS values. This suggests that cross-study comparability depends on standardization of both ROI definition and imaging protocol [28,29].

The single histopathological study offers compelling structural confirmation of lymphatic elements within the inner ear but is restricted to surgically treated advanced Ménière disease, thereby limiting extrapolation to physiological or early-stage conditions [5]. An interesting study on pediatric patients indicates that glymphatic alterations are already detectable in untreated congenital sensorineural hearing loss, suggesting that disrupted brain–ear fluid dynamics may represent an early and lifespan-spanning neurobiological substrate rather than a late epiphenomenon of aging [27]. On the other hand, the MRI-based, cognitive and audiological findings in adults reported by four authors suggested that glymphatic dysfunction may mediate the increasingly documented association between hearing loss [6,26], tinnitus [24,25] and cognitive decline. This mechanistic framework may help reconcile epidemiological data linking ARHL to neurodegenerative risk [30]. Furthermore, the potential reversibility of glymphatic impairment raises therapeutic implications, including pharmacological modulation of glymphatic pathways [31] and emerging surgical strategies targeting fluid dynamics [7]. While speculative at present, these findings may offer a preliminary translational framework linking auditory disorders with broader neurovascular and glymphatic processes. Importantly, these findings should not be interpreted as demonstrating a causal link between hearing loss and cognitive decline. The current evidence is cross-sectional and based largely on indirect MRI proxies; therefore, causality and directionality cannot be established. Bidirectional interactions and shared systemic confounding factors, including aging-related, vascular, inflammatory, and sleep-related mechanisms, remain plausible explanations [32].

Several limitations of this systematic review must be acknowledged. First, the number of eligible human studies remains small, precluding robust quantitative synthesis and limiting statistical power. Second, although statistical heterogeneity was low in the quantitative synthesis, clinical and methodological variability across populations and imaging protocols remains substantial. Third, most evidence derives from cross-sectional MRI studies using indirect glymphatic markers while the single histopathological investigation was conducted in advanced Ménière disease requiring surgery, introducing selection bias and restricting generalizability. Fourth, although core systematic review procedures were preserved, the absence of protocol registration reduced methodological transparency and reproducibility producing an increased risk of reporting bias. Finally, current literature is limited to case series and observational studies while prospective longitudinal studies evaluating temporal relationships between hearing loss, glymphatic dysfunction, and cognitive trajectories are currently lacking. Future research should prioritize multicenter prospective designs, standardized imaging protocols, and, where feasible, multimodal validation combining neuroimaging with molecular or histopathological correlates. Such efforts are essential to determine whether the observed associations represent epiphenomena or reflect a true pathophysiological pathway linking auditory and neurodegenerative processes. Further research should explore whether glymphatic dysfunction also plays a role in vertigo and dizziness disorders.

## 5. Conclusions

This systematic review and meta-analysis provides the first quantitative synthesis of human evidence linking auditory disorders to alterations in glymphatic function. Although based on a limited number of heterogeneous studies, the pooled data showed significantly reduced ALPS indices in patients with tinnitus, congenital SNHL, and age-related hearing loss compared with controls. These findings support the hypothesis that impaired central fluid dynamics may represent a shared pathophysiological substrate connecting inner ear pathology with cognitive vulnerability. However, according to GRADE criteria, the overall certainty of evidence remains low, as current data derive exclusively from cross-sectional observational studies using indirect MRI-based glymphatic proxies, while direct labyrinthine lymphatic assessment remains scarce. This limitation constrains causal inference and generalizability. Larger prospective studies integrating standardized imaging, audiological characterization, and longitudinal cognitive outcomes are warranted to clarify whether glymphatic dysfunction is merely associative or represents a modifiable therapeutic target.

## Figures and Tables

**Table 1:** Main characteristics and Risk of Bias of included studies.

Study	Design	RoB Instrument Used	Primary Limitation	Overall RoB
Xu 2024 [26]	Cross-sectional	NOS	Residual vascular/metabolic confounding	Low–Moderate
Zhang 2024 [5]	Surgical case-series	JBI	Case selection bias	Moderate
Sha 2024 [27]	Cross-sectional (pediatric)	NOS	Small sample size; manual ROI placement for DTI-ALPS measurement	Moderate
Du 2024 [24]	Cross-sectional	NOS	Manual ROI placement for DTI-ALPS measurement; potential residual confounding	Moderate
Du 2025 [25]	Cross-sectional	NOS	Phenotype heterogeneity; manual ROI placement for DTI-ALPS measurement	Moderate
Ye 2025 [6]	Cross-sectional	NOS	Manual ROI placement for DTI-ALPS measurement	Low–Moderate

Abbreviations: Newcastle–Ottawa Scale (NOS); Joanna Briggs Institute (JBI) Case series Tool; Region of Interest (ROI); Diffusion Tensor Image Analysis Along the Perivascular Space (DTI-ALPS).

**Table 2:** PICO-Based Evidence Synthesis of included studies.

Study	Population	Mean Age ± SD (Years)	Intervention/Exposure	Comparator	Outcomes	Key Findings
Xu 2024 [26]	ARHL and cognitive decline n = 30; ARHL n = 30; HCs n = 40	ARHL and cognitive decline: 63.20 ± 7.33; ARHL: 62.20 ± 7.05; HCs: 61.55 ± 3.72	MRI (DTI-ALPS), assessment of cognitive status	HCs and Between-group comparison	DTI-ALPS index; correlation with cognitive performance	Reduced ALPS index in ARHL patients, particularly in those with cognitive decline, supporting a link between hearing loss, impaired glymphatic function, and cognitive impairment
Zhang 2024 [5]	MD n = 21	60.67 ± 7.92	Histopathological examination of inner ear specimens	NA	Detection of lymphatic markers (D2-40, LYVE-1, PROX1, podoplanin); TEM ultrastructure	First direct structural demonstration of lymphatic capillaries in human inner ear pathology (MD), suggesting reactive lymphangiogenesis
Sha 2024 [27]	CSNHL n = 26; HCs n = 30	CSNHL: 4.56 ± 1.90; HCs: 4.63 ± 2.04	MRI (DTI-ALPS)	HCs	DTI-ALPS index	Children with CSNHL showed significantly lower ALPS index compared to controls, indicating early-life glymphatic dysfunction associated with hearing impairment
Du 2024 [24]	Tinnitus n = 50; HCs n = 50	Tinnitus: 50.38 ± 13.56; HCs: 48.76 ± 15.21	MRI (DTI-ALPS); assessment of cognitive status	HCs	DTI-ALPS index; correlation with tinnitus severity	Significantly reduced ALPS index in tinnitus patients compared to controls, suggesting impaired central glymphatic function associated with auditory pathology
Du 2025 [25]	TSD n = 29; TNSD n = 29; HCs n = 38	TSD: 63.62 ± 7.08; TNSD: 62.59 ± 6.85; HCs: 61.45 ± 3.79	MRI (CPV, EPVS, DTI-ALPS); assessment of cognitive status	HCs and Between-group comparison	DTI-ALPS index; CPV and EPVS; correlation with tinnitus severity and sleep quality	Central glymphatic dysfunction associated with auditory pathology, worsening in subject with sleep disorders; indirect evidence of auditory–glymphatic interaction
Ye 2025 [6]	ARHL n = 130; HCs n = 121	ARHL: 64.10 ± 3.43; HCs: 63.55 ± 3.49	MRI (CPV, EPVS, DTI-ALPS); inflammatory biomarkers (TNF-α, IL-1β, IL-6); assessment of cognitive status	HCs	DTI-ALPS index; CPV and EPVS; correlation and mediation with cognition	Hearing loss associated with systemic inflammation and impaired glymphatic function; suggests bidirectional neuro-auditory interaction

**List of Abbreviations**: Diffusion Tensor Image Analysis Along the Perivascular Space (DTI-ALPS); Age-Related Hearing Loss (ARHL); Choroid Plexus Volume (CPV); Enlarged Perivascular Spaces (EPVS); Healthy Controls (HCs); Interleukin-1 Beta (IL-1β); Interleukin-6 (IL-6); Ménière Disease (MD); Magnetic Resonance Imaging (MRI); NA (Not Applicable); Prospero Homeobox Protein 1 (PROX1); Transmission Electron Microscopy (TEM); Tumor Necrosis Factor Alpha (TNF-α); Tinnitus with Sleep Disturbance (TSD); Tinnitus without Sleep Disturbance (TNSD); Congenital Sensorineural Hearing Loss (CSNHL).

**Table 3:** Summary of Findings according to GRADE for the quantitative synthesis of DTI-ALPS index in auditory disorders.

Outcome	Participants (Studies)	Study Design	Effect Estimate	Certainty of Evidence	Reasons for Downgrading
DTI-ALPS index in auditory disorders vs. healthy controls	483 participants (5 studies)	Cross-sectional observational studies	SMD = −0.73 (95% CI −0.90 to −0.55)	Low	Downgraded for risk of bias (observational cross-sectional studies, manual ROI placement, residual confounding) and indirectness (heterogeneous auditory phenotypes and indirect MRI-based glymphatic proxies rather than direct inner-ear assessment)

## Data Availability

The raw data supporting the conclusions of this article will be made available by the authors on request.

## Supplementary Materials

The following supporting information can be downloaded at: https://www.mdpi.com/article/10.3390/medicina62050878/s1, Table S1: NOS-based assessment of observational studies; Table S2: JBI checklist assessment of case series.
