# Supplementary material for: Lymphatic and Glymphatic Alterations in Auditory Disorders: A Rapid Review-Informed Systematic Review and Meta-Analysis
Source: Medicina (Kaunas). 2026 May 3;62(5):878. doi: 10.3390/medicina62050878 (PMC13208798; doi:10.3390/medicina62050878)
Supplement: Supplementary file 1 [file medicina-62-00878-s001.zip › medicina-4210725-supplementary.pdf]

Supplementary Materials “Lymphatic and Glymphatic Alterations in Auditory Disorders: A Rapid Systematic Review and Meta-Analysis” Frosolini et al. 2026;. Item-level quality assessment and risk-of-bias appraisal of included studies

Table S1 NOS-based assessment of observational studies

Study	Study design	Selection: case definition / exposure ascertainment	Selection: representativeness of cases	Selection: selection of controls	Selection: definition of controls	Comparability: matching / adjustment	Outcome/Exposure assessment	Same assessment method for groups	Statistical reporting adequate	Main unmet criteria	Overall RoB
Du 2024	Cross-sectional case-control	Yes	Yes	Yes	Yes	Partial – matched for age, sex, education, but residual confounding possible	Yes	Yes	Yes	Residual confounding; manually placed DTI-ALPS ROIs	Moderate
Du 2025	Prospective cross-sectional case-control	Yes	Yes	Yes	Yes	Partial – matched for age, sex, education, but tinnitus subgroups introduce phenotype heterogeneity	Yes	Yes	Yes	Phenotype heterogeneity; manual ROI placement; residual confounding	Moderate
Sha 2024	Cross-sectional pediatric case-control	Yes	Partial – relatively small single-center pediatric sample	Yes	Yes	Partial – age/sex matched, but limited adjustment for additional confounders	Yes	Yes	Yes	Small sample; manual ROI placement; limited confounder control	Moderate
Xu 2024	Cross-sectional case-control	Yes	Yes	Yes	Yes	Yes – groups matched and analyses adjusted for age, gender, education	Yes	Yes	Yes	Residual vascular/metabolic confounding; manual ROI approach	Low–Moderate
Ye 2025	Cross-sectional observational study	Yes	Yes	Yes	Yes	Yes – controls matched; broader clinical exclusion criteria and mediation/correlation analyses performed	Yes	Yes	Yes	Manual ROI placement; cross-sectional design	Low–Moderate

Table S2 JBI checklist assessment of case series

Study	Clear inclusion criteria	Standard/reliable condition measurement	Valid identification methods	Consecutive inclusion	Complete inclusion	Demographics clearly reported	Clinical information clearly reported	Outcomes/follow-up clearly reported	Site demographics clearly reported	Statistical analysis appropriate	Main unmet criteria	Overall RoB
Zhang 2024	Yes	Yes	Yes	Unclear	Unclear	Yes	Yes	Yes	Yes	Limited / descriptive	Possible case-selection bias; modest sample; limited clarity on consecutive completeness	Moderate
